# The Effectiveness of Influenza Vaccination on Chronic Obstructive Pulmonary Disease with Different Severities of Airflow Obstruction

**DOI:** 10.3390/biomedicines9091175

**Published:** 2021-09-07

**Authors:** Hui-Chuan Chang, Shih-Feng Liu, Ying-Chun Li, Ho-Chang Kuo, Yun-Chyn Tsai, Min-Hui Chen

**Affiliations:** 1 Department of Respiratory Therapy, Kaohsiung Chang Gung Memorial Hospital, Kaohsiung 833, Taiwan; elaine11142@cgmh.org.tw (H.-C.C.); erickuo48@yahoo.com.tw (H.-C.K.); jane2793@cgmh.org.tw (Y.-C.T.); elisa2840@cgmh.org.tw (M.-H.C.); 2 Department of Internal Medicine, Division of Pulmonary and Critical Care Medicine, Kaohsiung Chang Gung Memorial Hospital, Kaohsiung 833, Taiwan; 3 Medical Department, College of Medicine, Chang Gung University, Taoyuan 333, Taiwan; 4 Institute of Health Care Management, National Sun Yat-sen University, Kaohsiung 833, Taiwan; ycli@mail.nsysu.edu.tw; 5 Department of Pediatrics, Kaohsiung Chang Gung Memorial Hospital, Kaohsiung 833, Taiwan

**Keywords:** influenza vaccination, COPD, effectiveness, airflow obstruction, hospital utilization, emergency utilization, respiratory failure

## Abstract

This retrospective study included COPD patients who attended our medical center between January and October 2018, and analyzed the outcomes of their influenza vaccination, including medical visits, hospitalization, medical expenses, and the incidence of respiratory failure. Airflow limitation was stratified according to GOLD guidelines. Overall, 543 COPD patients were enrolled, including 197, 113, 126, and 107 mild, moderate, severe, and very severe patients, respectively. Of all the participants, 238 received an influenza vaccination (43.8%), which significantly reduced hospital utilization for moderate (odds ratio [OR] 0.22, 95%CI 0.09–0.51), severe (OR 0.19, 95%CI 0.08–0.44), and very severe patients (OR 0.15, 95%CI 0.05–0.5) compared to mild patients (OR 0.51, 95%CI 0.2–1.26); reduced emergency department utilization for moderate (OR 0.33, 95%CI 0.14–0.77), severe (OR 0.22, 95%CI 0.10–0.52), and very severe patients (OR 0.30, 95%CI 0.10–0.88) compared to mild patients (OR 0.64, 95%CI 0.30–1.37); and reduced the occurrence of respiratory failure for moderate (OR 0.20, 95%CI 0.06–0.68), severe (OR 0.40, 95%CI 0.16–0.98), and very severe patients (OR 0.36, 95%CI 0.15–0.82) compared to mild patients (OR 0% CI 0.14–3.20). Influenza vaccination is more effective in COPD patients with moderate, severe, and very severe airflow obstruction than in those with mild obstruction with respect to hospital utilization, emergency department utilization, and respiratory failure.

## 1. Introduction

Chronic obstructive pulmonary disease (COPD) is a progressive disease that worsens over time. However, it can be kept under control with medication [1]. It is characterized by persistent respiratory symptoms and airflow limitation, which are caused by abnormalities in the airways and/or alveoli, usually due to heavy exposure to toxic particles or gases, and is affected by host factors [2]. Airflow limitation is usually measured by spirometry because this is the most widely used and reproducible lung function test. Chronic and progressive dyspnoea are the most typical symptoms of COPD [3]. These symptoms may be under-reported by patients, they may vary from day to day, and they may be present for many years before the development of airflow limitation [4]. A person may seek medical help due to chronic respiratory symptoms or due to the exacerbation of acute respiratory symptoms. A COPD exacerbation is defined as an acute worsening of respiratory symptoms leading to additional treatment. It is usually associated with increased airway inflammation, increased mucus production, and significant gas trapping. Exacerbations of COPD can be caused by many factors. The most common cause is respiratory infection. Acute exacerbations are mainly caused by respiratory viral infections, but bacterial infections and environmental factors, such as pollution and ambient temperature, may also cause acute exacerbations [5].

Influenza is a disease with a global impact, causing huge morbidity and mortality every year. It mainly infects the respiratory tract and causes a wide range of diseases, from asymptomatic infections to fulminant primary viral pneumonia and secondary bacterial pneumonia. The severity of infection depends on the virus strain and many host factors, mainly age and the presence of comorbidities, such as cardiopulmonary disease. In particular, in patients with COPD, the morbidity caused by influenza infections is much higher than in those without COPD [6]. As research has shown, COPD admission rates were associated with influenza activity [7]; and cases of COPD with pandemic influenza infection were significantly associated with ICU admission and death [8]. In addition, the viral–bacterial co-infection in the lungs involves a synergy between the influenza virus and streptococcus pneumoniae [9]. The severity of pneumonia in patients co-infected with the influenza virus and bacteria was significantly higher than in those infected with bacteria or the influenza virus alone [10]. Therefore, the influenza vaccination is recommended for patients with COPD [2,11]. Research has shown that influenza vaccination can reduce the incidence of pneumonia in COPD patients, as well as reducing the rate of hospitalization and mortality [12], and it can also effectively prevent COPD patients from being admitted to hospital due to acute exacerbations [13]. Further, influenza vaccination can reduce the number of outpatient visits, as well as the rate of respiratory failure and mortality, as described in a systemic analysis [14]; it can also reduce influenza-related hospitalization and mortality, and influenza-induced complications, as mentioned in a Canadian secondary data analysis [15]. However, another study showed that there was no significant difference in the effectiveness of influenza vaccination between patients with and without COPD, although it did reduce the rate of serious diseases in high-risk patients [16]. An 18-week study also reported that the vaccinated group had more respiratory symptoms than the placebo group [17]. This may be due to greater heterogeneity among COPD patients, or because they have different severities of airflow obstruction, or even because they are often elderly people with other chronic diseases, which may lead to a reduced immune response to vaccination [18]. Additionally, a randomized controlled study showed that influenza vaccination was highly effective in the prevention of influenza-related acute respiratory illness, regardless of the severity of COPD [19].

To date, there are few data about whether influenza vaccination has different effects on COPD patients with variable severities of airflow obstruction. This study explores the effectiveness of influenza vaccination on COPD patients with different severities of airflow obstruction.

## 2. Materials and Methods

### 2.1. Design

**Figure 1:**
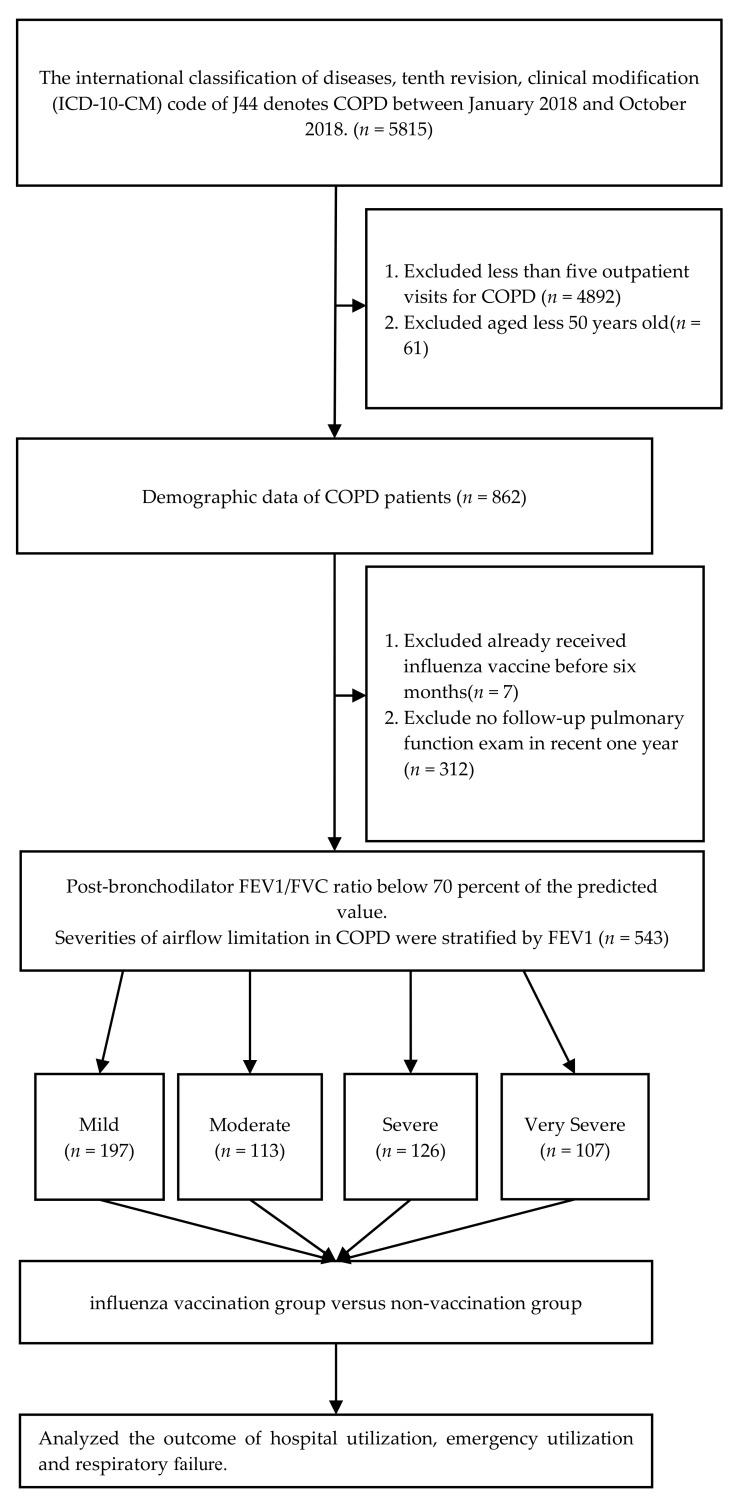
Flowchart of study design and patient enrolment.

This was a retrospective study designed to analyse patients diagnosed with COPD from January to October 2018 in the database of Kaohsiung Chang Gung Memorial Medical Center, in order to explore the effectiveness of influenza vaccination on groups with different levels of airflow obstruction. We tracked these participants until 30 September 2019, and analyzed the incidents related to respiratory tract infections, pneumonia, acute exacerbations, and influenza, including the number of outpatient and emergency medical visits, the number of hospitalization days, medical expenses, and the occurrence of respiratory failure (Figure 1).

### 2.2. Study Participants

The inclusion criteria was the main diagnosis code of the International Classification of Diseases, Tenth Revision, Clinical Modification (ICD-10) J44 and a record of more than five outpatient visits in 2018. We excluded those who did not meet the COPD diagnostic criteria on pulmonary function tests, those patients who made fewer than five outpatient visits per year, those who were younger than 50 years old, those who were vaccinated against influenza within 6 months before enrolment, and those who were not followed up for pulmonary function tests within the previous year. The lung function criterion for airflow limitation for COPD patients was designed according to GOLD guidelines: a percentage predicted value for post-bronchodilator FEV1/FVC ratio lower than 70%, diagnosed as COPD, and then classified into four severities of airflow limitation by FEV1: Mild COPD (FEV1 > 80%), Moderate COPD (FEV1 50–80%), severe COPD (FEV1 30–50%), and very severe COPD (FEV1 < 30%) [2]. We selected COPD participants who had made than five outpatient visits per year. This was mainly based on the findings of a previous 6-year observational study, which observed that the average annual number of outpatient visits for COPD patients was 4.9 times per year [20]; and the fact that the stable COPD patients in our hospital return to outpatient visit for follow-up at an average rate of approximately once every 2 months. A total of 5815 patients met ICD-10-CM Diagnosis Code J44.0 and 5272 patients were excluded. Subsequently, 543 patients fulfilled the inclusion and exclusion criteria and were included in this study (Figure 1). 

### 2.3. Influenza Vaccines

Two kinds of influenza vaccine were used. One was AdimFlu-S (Adimmune Corporation, Taichung, Taiwan), a purified, trivalent, deactivated spilt-virus vaccine. One dose of 0.5 mL of this vaccine contains A/Michigan/45/2015 (H1N1), A/ Hong Kong/4801/2014 (H3N2), and B/ Brisbane/60/2008-like virus. The other was VAXIGRIP (Sanofi Pasteur, France), another purified, trivalent, deactivated spilt-virus vaccine. One dose of 0.5 mL of vaccine contains A/Michigan/45/2015 (H1N1), A/Singapore/INFIMH-16-0019/2016 (H3N2), and B/Brisbane/60/2008-like strain. The vaccinated participants received either the AdimFlu-S or the VAXIGRIP vaccine.

### 2.4. Clinial Variables

Demographic data, including gender, age, smoking status, charlson comorbidity index (CCI), influenza vaccination status, and a complete medical history including the number of exacerbations and emergency department visits, hospitalizations, medical expenditure, occurrences of respiratory failure and current medication, were recorded during the study period.

### 2.5. Statistics

Continuous variables are expressed as mean ± SD, and categorical variables are expressed as absolute values and as percentages. The participants with COPD were divided into four major groups, according to the severity of their expiratory airflow (FEV1). The variables included whether or not the participants had received an influenza vaccination, the number of emergency room visits participants had made, the length of their hospital stay, medical expenses, and respiratory failure occurrence. The performance of chi-square independence tests in categorical variables and one-way analysis of variance (ANOVA) was carried out in continuous variables. The cumulative incidence of outcomes, including hospitalizations, medical expenses, and relative influenza vaccination with each classification of COPD, was studied using multivariate logistic regression. To relate the number of hospitalization and emergency department visits and relative influenza vaccination to each classification of COPD, we used Cox negative binomial regression analysis. Data analysis was conducted using STATA Version 12 (College Station, TX, USA). A 2-sides *p*-value of <0.05 was considered significant.

## 3. Results

### 3.1. Patients’ Characteristics

Table 1 shows the characteristics of the study participants. There were 543 patients enrolled in the final analyses, with an average age of 71.77 ± 9.26 years. There were 453 males (83.4%) and 357 smokers (65.75%). There were 197 mild COPD patients, 113 moderate COPD patients, 126 severe COPD patients, and 107 very severe COPD patients. Out of the total number of study participants, 238 had received an influenza vaccination (43.8%). Males had a higher prevalence in each group. Among each group, the number of participants who were not vaccinated against influenza was higher than the number of participants who were. Smoking status showed a higher proportion of smokers than non-smokers among each group and, in addition, successful smoking cessation was higher in proportion than non-successful-smoking cessation in the moderate, severe, and very severe COPD groups, although not in the mild COPD group. The patients with CCI ≥ 2 had a higher proportion than those with CCI = 1 and CCI = 0 in each group. No serious adverse effects or deaths after influenza vaccination were recorded. 

### 3.2. Effectiveness of Influenza Vaccination Measured by Hospital Utilization, Hospitalization Days, and Hospitalization Expenditure for COPD Patients with Different Severities of Airflow Obstruction

**Figure 2:**
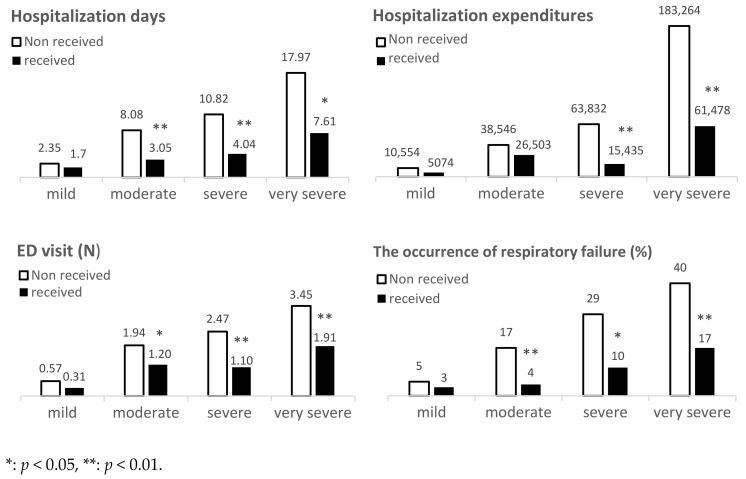
Effectiveness of influenza vaccination measured by hospitalization days, hospitalization expenditure, number of emergency department visits and occurrence of respiratory failure in COPD patients with different severities of airflow obstruction.

Table 2 and Figure 2 show that influenza vaccination was associated with medical utilization among COPD patients with different severities of airflow obstruction. For hospital utilization, the influenza vaccination group was lower in moderate, severe, and very severe COPD patients than the non-vaccination group (*p* < 0.001), but there was not a significant difference in mild COPD patients (*p* = 0.137). There were fewer hospitalization days noted among moderate (*p* < 0.001), severe (*p* = 0.002), and very severe COPD patients (*p* = 0.017) in the vaccination group than in the non-vaccination group, but there was not significant difference among mild COPD patients (*p* = 0.582). The total hospitalization expenditure of the influenza vaccination group was lower among severe (*p* = 0.001) and very severe COPD patients (*p* = 0.006) than in the non-vaccination group, but there was not significant difference among mild (*p* = 0.338) and moderate COPD patients (*p* = 0.440).

### 3.3. Effectiveness of Influenza Vaccination Measured by Emergency Department Utilization and Number of Visits in COPD Patients with Different Severities of Airflow Obstruction

Emergency department utilization in the influenza vaccination group was lower among moderate (*p* = 0.006), severe (*p* < 0.001), and very severe COPD patients (*p* = 0.021) than in the non-vaccination group, but there was no significant difference among mild patients (*p* = 0.408). For emergency department visit numbers, the influenza vaccination group was lower among moderate (*p* = 0.049), severe (*p* = 0.009), and very severe COPD patients (*p* = 0.004) than the non-vaccination group, but there was no significant difference among mild patients (*p* = 0.210) (Table 2) (Figure 2).

### 3.4. Effectiveness of Influenza Vaccination Measured by Occurrence of Respiratory Failure in COPD Patients with Different Severities of Airflow Obstruction

The influenza vaccination group had a significantly lower risk of respiratory failure among moderate (*p* = 0.008), severe (*p* = 0.041), and very severe COPD patients (*p* = 0.006) than non-vaccination group, but there was no significant difference among mild patients (*p* = 0.574) (Table 2) (Figure 2).

### 3.5. Adjusted Odds Ratio of Medical Utilization According to Influenza Vaccination Status among COPD Patients with Different Severities of Airflow Obstruction

**Figure 3:**
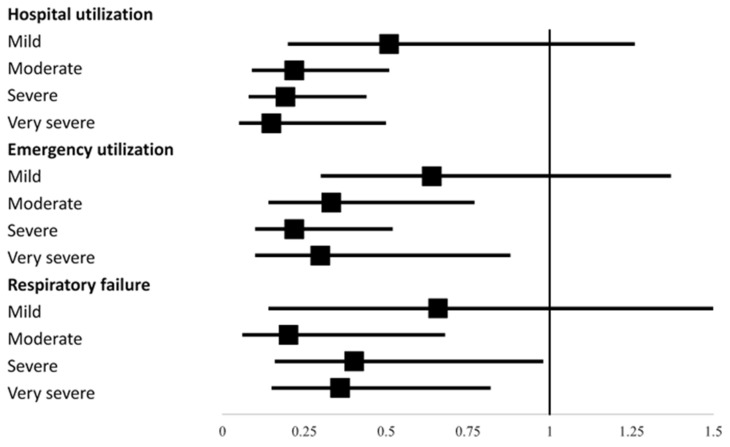
Adjusted odds ratio and 95% confidence interval of the likelihood of medical utilization according to influenza vaccination status among COPD patients with different severities of airflow obstruction.

Table 3 shows the probability of using medical resources, such as hospital utilization, emergency utilization and the occurrence of respiratory failure, according to influenza vaccination status. Influenza vaccination significantly reduced the odds ratio of hospital utilization, emergency utilization and the occurrence of respiratory failure among the moderate, severe and very severe COPD patients, but there was no significant difference among mild COPD patients (Figure 3).

## 4. Discussion

This retrospective study shows that the influenza vaccination is safe and effective for COPD patients. Compared to patients with mild obstruction, influenza vaccination is more effective for patients with very severe, severe and moderate airflow obstruction. The number of emergency department visits, hospitalization utilization, length of hospital stay, and the occurrence of respiratory failure are significantly reduced in these patients. Compared to patients with moderate and mild obstructive airflow obstruction, COPD patients with severe and very severe airflow obstruction also had significantly reduce hospitalization costs in the vaccination group.

Influenza vaccination is beneficial for COPD patients for the following reasons. First, exacerbations of COPD have a negative impact on health status, hospitalization and readmission rates, and disease progression [2,21]. There are many causes of acute COPD exacerbations, including viral infections, bacterial infections, air pollution, temperature, etc [5,22,23]. Acute exacerbations caused by viruses are often more serious, last for a long time, and hospitalization is longer [24]. Influenza vaccination can efficiently prevent influenza-related acute respiratory disease in COPD patients with any airflow severity and it is therefore strongly recommended for all COPD patients [19]. It can also prevent the occurrence of influenza, thereby reducing acute exacerbations of COPD, and avoiding the subsequent adverse developments caused by acute exacerbations in patients. 

Second, influenza infections can change the host’s immunity to bacteria and co-infection and secondary bacterial infections often occur after viral infections [9,25,26,27,28,29]. Influenza is the main cause of epidemics and pandemic infections. Concurrent/secondary bacterial infections further increase the morbidity and mortality of influenza infections. Streptococcus pneumoniae, Haemophilus influenzae, and Staphylococcus aureus are reported as the most common bacteria species [25]. Influenza can suppress the immune response to pneumococcus infection and cause the up-regulation of pneumococcal adhesion molecules, leading to an increase in pneumococcal load and a decrease in survival [27,28]. Therefore, secondary pneumococcal pneumonia may lead to increased respiratory tract morbidity and mortality, especially in COPD with influenza infection [27,28]. The clinical course and microbiology of severe influenza and bacterial co-infection was described by McCullers et al. [28]. Bacterial co-infection usually occurs within the first 6 days of influenza infection and its presentation is similar to influenza infection alone, but the risk of death is increased [10,28]. Influenza vaccination can prevent the occurrence of influenza and reduce the occurrence of upper or lower respiratory tract viral and bacterial coinfections in COPD patients, and thus reduce the occurrence of acute exacerbations or severe pneumonia. 

Third, in addition to the prevention of upper respiratory tract infections, influenza causes many deaths from lower respiratory tract infections every year [30]. Influenza vaccination can also reduce influenza-associated acute lower respiratory tract infection in the elderly and in hospitalized patients [31,32]. Influenza vaccination is more effective in COPD patients with low FEV1 than in patients with higher FEV1. The possible reasons for this are as follows. First, bacterial colonization is an important factor in COPD disease progression. The decrease of FEV1 is often correlated with sputum IL-8. The increase of bacterial load in the airways and the change of species are often correlated with inflammation of the respiratory tract and the deterioration of lung function. FEV1 < 50% vs. FEV1 > 50% of COPD patients have a higher rate of sputum and potentially pathologic microorganisms, which are more likely to cause more severe acute exacerbations [33]. The preceding viral infection reduces the host’s immunity to bacteria, and co-infection with the virus at the same time increases the severity of the disease. Second, severe airflow limitation is one of the risk factors for acute COPD exacerbation [34]. COPD is a multi-dimensional disease [35]. The risk factors for acute exacerbation include exacerbation frequency, age, FEV1 level, BMI, the degree of dyspnoea, exertional capacity, nutritional status, lifestyle, length of disease course, comorbidities, etc [34,36]. Although FEV1 cannot represent the overall severity of COPD disease, FEV1 is still an important clinical reference index. When FEV1 is in decline, it is clinically worsening, and it will have a greater impact on lower-FEV1 patients. It is reasonable to assume that the effect of influenza vaccination in patients with poor FEV1 is greater than in COPD patients with higher FEV1.

In a study by Wongsurakiat et al., it was observed that influenza vaccination can prevent influenza-related acute respiratory disease regardless of the severity of COPD, but it does not prevent other ARIs unrelated to influenza. [19]. In their study, hospitalization and mechanical ventilator use were lower in the vaccinated group than in the non-vaccinated group but the difference was not statistically significant. Additionally, no mechanical ventilator use occurred in the vaccinated group, but there are three cases noted in the non-vaccinated group. Two patients were hospitalized in the vaccinated group and five were hospitalized in the non-vaccinated group. The 124 participants, including 62 cases in the vaccinated group and 62 in the non-vaccinated group, were divided into three different levels of airflow in the study: FEV1 > 70% predicted; FEV1 50–69% predicted; and FEV1 < 50% predicted. Therefore, there were about 16–23 cases in each subgroup. If the number of enrolled patients increases, hospitalization and mechanical ventilator use may achieve statistical differences. Although our study showed that influenza vaccination can significantly reduce hospital utilization and the occurrence of respiratory failure in COPD patients with moderate, severe, and very severe air-flow obstruction, a larger prospective study is still needed in order to confirm our findings. Influenza vaccine also had a protective effect that reduced morbidity and mortality in patients with cardiovascular disease [37,38]. COPD is associated with significant concomitant chronic diseases, which increase its morbidity and mortality. It implies that influenza vaccine can reduce non-influenza-related hospitalization and mortality in COPD. Additionally, the FEV1 cut-off value of Wongsurakiat et al’s grouping was different from the one used in our research and may yield different results. 

This study had some limitations. First, this was a retrospective study rather than a prospective study. Second, we excluded cases of influenza vaccination within the 6 months before the patient was enrolled, mainly to avoid the protective effect of the previous influenza vaccination. However, whether 6 months is sufficient or not is still subject to confirmation. Third, we excluded patients with less than five outpatient visits a year, mainly based on the 6-year study of Rhee et al. [20] and the consideration that the average number of return visits among our stable patients is about 2 months. There may be some very stable COPD patients, who come back once every 3 months but only come back four times a year; these would have been excluded from the study. Fourth, we analyzed only COPD-related events, including the number of emergency visits, the length of hospital stay, hospitalization medical expenses, and respiratory failure. We did not only include events related to influenza-related acute respiratory disease.

## 5. Conclusions

This study shows that influenza vaccination can reduce hospital utilization, emergency utilization, and the occurrence of respiratory failure in COPD patients. Moreover, influenza vaccination is more effective for COPD patients with moderate, severe, and very severe airflow obstruction than for those with mild obstruction.

## Figures and Tables

**Table 1:** Demographic characteristics of the study population.

Severity Variables	Mild		Moderate		Severe		Very Severe	
Observation	197		113		126		107	
Age	71.37(8.47)		71.18(8.50)		70.73(9.29)		72.08(10.07)	
Gender								
Male	171	86.80%	87	76.99%	110	87.30%	83	77.57%
Female	26	13.20%	26	23.01%	16	12.70%	24	22.43%
Vaccination								
Yes	93	47.21%	51	45.13%	49	38.89%	45	42.06%
No	104	52.79%	62	54.87%	77	61.11%	62	57.94%
Smoking Status								
Ex-smoker	123	62.44%	71	62.83%	96	76.19%	67	62.62%
Non-smoker	74	37.56%	42	37.17%	30	23.81%	40	37.38%
Successful smoking Cessation								
Yes	57	46.34%	44	61.97%	51	53.13%	48	71.64%
No	66	53.66%	27	28.03%	45	46.88%	19	28.36%
Charlson comorbidity index								
0	56	28.43	20	17.70	21	16.67	18	16.82
1	49	24.87	24	21.24	41	32.54	15	14.02
≥2	92	46.70	69	61.06	64	50.79	74	69.16

**Table 2:** Effectiveness of influenza vaccination measured by rates of medical utilization in COPD patients with different severities of airflow obstruction.

Severity Variables	Mild			Moderate			Severe			Very Severe		
Influenza Vaccination	Non-Received	Received	*p* Value	Non-Received	Received	*p* Value	Non-Received	Received	*p* Value	Non-Received	Received	*p* Value
Hospital utilization (%)	18.27	10.75	0.137	75.81	41.18	<0.001	79.22	40.82	<0.001	88.71	62.22	0.001
Hospitalization, days (SD)	2.35 (8.42)	1.70 (8.00)	0.582	8.08 (7.86)	3.05 (4.40)	<0.001	10.82 (14.32)	4.04 (5.99)	0.002	17.97 (23.19)	7.61 (11.69)	0.017
Hospitalizations expenditures mean ± SD	10,554 (51,737)	5074 (19,650)	0.338	38,546 (56,437)	26,503 (104,248)	0.440	63,832 (100,230)	15,435 (24,015)	0.001	183,264 (282,776)	61,478 (75,215)	0.006
ED utilization (%)	23.08	18.28	0.408	77.42	52.94	0.006	77.92	46.94	<0.001	88.71	71.11	0.021
ED visit, N (%)	0.57 (1.80)	0.31 (1.00)	0.210	1.94 (2.25)	1.20 (1.56)	0.049	2.47 (3.37)	1.10 (1.61)	0.009	3.45 (3.13)	1.91 (1.93)	0.004
Respiratory failure occur (%)	4.81	3.23	0.574	27.42	7.84	0.008	37.66	20.41	0.041	64.52	37.38	0.006

**Table 3:** Adjusted odds ratio of the likelihood of medical utilization according to influenza vaccination status among COPD patients with different severities of airflow obstruction.

	Hospital Utilization		Emergency Utilization		Respiratory Failure	
	OR(95%CI)	*p* value	OR(95%CI)	*p* value	OR(95%CI)	*p* value
Mild	0.51(0.2–1.26)	0.133	0.64(0.30–1.37)	0.407	0.66(0.14–3.20)	0.571
Moderate	0.22(0.09–0.51)	0.000 *	0.33(0.14–0.77)	0.006	0.20(0.06–0.68)	0.006
Severe	0.19(0.08–0.44)	0.000 *	0.22(0.10–0.52)	0.000 *	0.40(0.16–0.98)	0.037
Very severe	0.15(0.05–0.5)	0.001	0.30(0.10–0.88)	0.022	0.36(0.15–0.82)	0.006

*: *p* < 0.001.

## Data Availability

The data generated and analyzed in this study are included in the article.

## Abbreviations

COPD	Chronic Obstructive Pulmonary Disease
GOLD	Global Initiative for Chronic Obstructive Lung Disease
FEV1	Forced expiratory volume during the first second
FVC	Forced Vital Capacity
